# Supplementary material for: Cross-sectional relationship among different anthropometric parameters and cardio-metabolic risk factors in a cohort of patients with overweight or obesity
Source: PLoS One. 2020 Nov 5;15(11):e0241841. doi: 10.1371/journal.pone.0241841 (PMC7644056; doi:10.1371/journal.pone.0241841)
Supplement: Attachment — Submitted filename: Response to Reviewers.docx [file pone.0241841.s001.docx]

**Answers to Reviewer #1**

Firstly, we would like to thank the reviewer for having carefully reviewed our manuscript and for the useful advice given.

**1) The authors conclude that “…BMI and WC seem to be the essential anthropometric parameters for use in clinical practice to quantify the cardio-metabolic risk…”. However, the current study is only observational and exploited pair-wise associations – yet, age- and gender-corrected - between the anthropometric and cardiovascular parameters. How did the authors identify BMI and WC (among the investigated parameters) as the “essential ones”? Analyses and comparison of the strength of associations with the clinical parameters could be performed to support this conclusion.**

We totally agree with the reviewer. For this purpose, we extensively modified the text, in the light of the new analysis performed. Please read the next points, in which all the changes made are explained

**2) The authors have assessed pairwise correlations with clinical parameters which are associated with incidence of future cardiovascular events. It would be interesting to know whether these anthropometric parameters also associate with the cardiovascular risk of these patients computed using standardized algorithms (e.g. FRS, SCORE, “Progetto Cuore”). Moreover, the individuation of the anthropometric parameter with the strong association (thus, potentially more relevant for clinical application) would significantly improve the findings of the authors.**

**3) Is there collinearity among the tested parameters? If collinearity is not relevant, the authors could also consider enter all the anthropometric parameters into the regression models (also with a stepwise approach) to verify whether some of them could independently associate with the clinical parameters (and/or the estimated cardiovascular risk, see point #2).**

2-3. For this purpose, we estimated cardiovascular risk by the SCORE Risk Charts and we combined all the anthropometric parameters into the regression models with a stepwise approach to verify whether some of them could be independently associated with the SCORE Risk Charts. We used these charts because they are the most commonly used, and validated in European populations. We also decided to add and discuss the new results of this analysis in our manuscript (see Result section lines 193-197 and Discussion section lines 210-211 and 213-219).

**4) Which is the percentage of patients fulfilling the diagnostic criteria for Metabolic Syndrome and Metabolically healthy obesity? Would the association still be significant in these two subpopulations?**

4. The prevalence of Metabolic Syndrome was 36.9% and prevalence of MHO (obese subject without Metabolic Syndrome) was 48.3%. We have also carried out the analyses in the sub-groups you mentioned. Please find the results shown at the end of this document (Table rev1a and Table rev1b)

**5) ll.87-88: HOMA-IR and 25-OH-VitD are included among the parameters associated with CV risk. A reference should be included here**.

5. We agree with the reviewer. Two references have been inserted:

- regarding HOMA-IR, in line 86 (17) *Ormazabal V, Nair S, Elfeky O, Aguayo C, Salomon C, Zuñiga FA. Association between insulin resistance and the development of cardiovascular disease. Cardiovasc Diabetol. 2018;17(1):122. Published 2018 Aug 31. doi:10.1186/s12933-018-0762-4*
- regarding Vitamin D, in line 87 (18) *Muscogiuri G, Annweiler C, Duval G, et al. Vitamin D and cardiovascular disease: From atherosclerosis to myocardial infarction and stroke. Int J Cardiol. 2017;230:577-584. doi:10.1016/j.ijcard.2016.12.053*

**Table rev1a.** Univariate linear regression models of SCORE and anthropometric parameters in patients with Metabolic Syndrome.

| Parameters ^*^ | **SCORE ^§^** | | |  |  |
| --- | --- | --- | --- | --- | --- |
|  | *β* | *p-value* | *C.I. (95%)* |  | |
|  |  |  |  |  | |
| Neck (cm) | 0.03 | 0.20 | -0.01 to 0.07 |  | |
| BMI (kg/m²) | -0.04 | 0.001 | -0.07 to -0.02 |  | |
| Waist (cm) | -0.005 | 0.34 | -0.01 to 0.005 |  | |
| Waist to Height Ratio | -2.02 | 0.02 | -3.70 to -0.34 |  | |
|  |  |  |  |  | |

^§^ Poisson regression Model.

^*^ Abbreviations: β, coefficient; C.I., Coefficient Interval at 95%; Systematic COronary Risk Evaluation (SCORE); FLI, BMI, Body Mass Index; BMI, Body Mass Index.

**Table rev1b.** Univariate linear regression models of SCORE and anthropometric parameters in MHO patients

| Parameters ^*^ | **SCORE ^§^** | | |  |  |
| --- | --- | --- | --- | --- | --- |
|  | *β* | *p-value* | *C.I. (95%)* |  | |
|  |  |  |  |  | |
| Neck (cm) | 0.06 | 0.04 | 0.002 to 0.113 |  | |
| BMI (kg/m²) | -0.14 | <0.001 | -0.20 to -0.08 |  | |
| Waist (cm) | 0.005 | 0.53 | -0.01 to 0.02 |  | |
| Waist to Height Ratio | 0.26 | 0.84 | -2.32 to 2.84 |  | |
|  |  |  |  |  | |

^§^ Poisson regression Model.

^*^ Abbreviations: β, coefficient; C.I., Coefficient Interval at 95%; Systematic COronary Risk Evaluation (SCORE); BMI, Body Mass Index; BMI, Body Mass Index.

**Answers to Reviewer #2**

Firstly, we would like to thank the reviewer for having accurately reviewed our paper and for giving useful advices.

Major issues:

**1. The use of anthropometric indices of body fat distribution to identify healthy individuals at increased cardiometabolic risk has been widely evaluated in a range of studies, most of them cross-sectional, many significantly larger than this one, and collectively spanning a range of ethnic groups. A subset of these studies are referenced in this paper. Many of these studies have demonstrated that measurement of waist circumference, waist-hip ratio or weight-height ratio affords superior predictive value than BMI alone. I am not therefore convinced that this study adds usefully to the existing literature.**

We appreciate the frank comment. However, at variance with most of the other papers, we simultaneously examined 4 anthropometric parameters, and NC had the strongest association with SCORE. In addition, this study was performed in a specific population of people with overweight or obesity, none of which was taking any kind of drug. The sum of these four aspects makes this study quite original.

**2. Given the question being asked and the clinical context in which anthropometric measurements are used, the use of receiver operating characteristic (ROC) analyses would be appropriate to evaluate the discriminatory power of BMI, WC, WtHR and NC.**

Unfortunately, we cannot proceed with the ROC analysis because of the design of our study, since we do not have a dichotomic variable as outcome.

**3. More information should be presented about the study participants. What was the ethnic composition? What was the age range? Indeed, it is not clearly stated that all participants were adults. What was the BMI range? For many of these parameters, simply reporting mean and SD is insufficient and makes it difficult have confidence in the statistical approach.**

We have added ranges to Table 1. Moreover, we have added the ethnic composition in the methods section (line 98, “all Caucasian”) and the range information you requested in the Results section (lines 168-170) and Table 1 “Table 1 shows the general, anthropometric, hormone, metabolic and routine biochemical characteristics of the enrolled population. As reported in the new text, our study population consisted of 31% men, mean age was about 40 years (range 14-70 years) and mean BMI was 34 kg/m^2^ (range 25-64.4)”

Minor issues:

**1. Graphical representations of the data/regression models would enhance the manuscript.**

Unfortunately, we cannot display graphic output of the analysis since they are linear regression models.

**2. Depending on the BMI distribution of the participants, it would be interesting to see if the models hold up at the higher BMI ranges (e.g. BMI >30).**

Below we show the table that explains the models considering only those subjects with a BMI over 30. All the models hold up, except those concerning inflammation parameters. The table (Table rev2) is shown at the end of this document.

**3. The male/female imbalance in this study is unexpected. The authors have not commented on potential reasons for this. There is an opportunity here to repeat the analyses in males vs females and see if any differences arise.**

We have added some comments on this imbalance in the final part of the discussion section (lines 263-266): “Two limitations of our study are the imbalance between the number of men and women and the broad age range of the study population. These could be due to the fact that the subjects spontaneously presented to our clinic for reasons of excessive weight, thus representing a selection bias”

Moreover, all association models were adjusted for gender. This means that all associations were estimated ceteris paribus of gender.

**4. There are some sections, particularly in the discussion, in which the wording could be improved to improve clarity.**

Following your advice, we have proceeded to have the manuscript again corrected by our native English speaker collaborator.

**Table rev2.** Multivariate regression models ^#^ between continuous ^§^ and categorical ^ψ^ variables and anthropometric parameters in patients with BMI>30.

|  | **Neck (cm)** | | |  | **BMI (Kg/m^2^)** | | |  | **Waist (cm)** | | |  | **Waist to Height Ratio** | | | |
| --- | --- | --- | --- | --- | --- | --- | --- | --- | --- | --- | --- | --- | --- | --- | --- | --- |
| Parameters ^*^ | *β* | *p-value* | *C.I. (95%)* |  | *β* | *p-value* | *C.I. (95%)* |  | *β* | *p-value* | *C.I. (95%)* |  | *β* | *p-value* | *C.I. (95%)* | |
|  |  |  |  |  |  |  |  |  |  |  |  |  |  |  |  | |
| *Continuous Variables ^§^* |  |  |  |  |  |  |  |  |  |  |  |  |  |  |  | |
| DBP (mmHg) | **0.35** | **0.001** | **0.13 to 0.57** |  | **0.13** | **0.02** | **0.02 to 0.25** |  | **0.10** | **0.001** | **0.04 to 0.14** |  | **10.18** | **0.01** | **2.47 to 17.89** | |
| SBP (mmHg) | **0.52** | **0.001** | **0.21 to 0.82** |  | **0.37** | **<0.001** | **0.21 to 0.53** |  | **0.17** | **<0.001** | **0.10 to 0.24** |  | **20.60** | **<0.001** | **9.53 to 31.64** | |
| Total Cholesterol (mg/dL) | 0.40 | 0.32 | -0.39 to 1.19 |  | -0.05 | 0.82 | -0.49 to 0.39 |  | -0.001 | 0.99 | -0.19 to 0.19 |  | 4.42 | 0.77 | -25.35 to 34.20 | |
| Triglyceride (mg/dL) | **3.55** | **<0.001** | **2.20 to 4.90** |  | **2.02** | **<0.001** | **1.30 to 2.75** |  | **0.85** | **<0.001** | **0.54 to 1.17** |  | **117.65** | **<0.001** | **68.42 to 166.88** | |
| FBG (mg/dL) | **0.57** | **<0.001** | **0.29 to 0.84** |  | **0.20** | **0.01** | **0.04 to 0.35** |  | **0.10** | **0.003** | **0.03 to 0.16** |  | **11.63** | **0.02** | **1.44 to 21.82** | |
| HDL (mg/dL) | **-0.88** | **<0.001** | **-1.13 to -0.64** |  | **-0.33** | **<0.001** | **-0.47 to -0.20** |  | **-0.16** | **<0.001** | **-0.22 to -0.10** |  | **-21.21** | **<0.001** | **-30.34 to -12.07** | |
| Insulin (mg/dL) | **1.83** | **<0.001** | **1.46 to 2.21** |  | **1.11** | **<0.001** | **0.91 to 1.31** |  | **0.43** | **<0.001** | **0.34 to 0.52** |  | **57.55** | **<0.001** | **43.66 to 71.44** | |
| HOMA-IR | **0.47** | **<0.001** | **0.37 to 0.57** |  | **0.27** | **<0.001** | **0.22 to 0.32** |  | **0.10** | **<0.001** | **0.08 to 0.13** |  | **14.00** | **<0.001** | **10.41 to 17.58** | |
| LDL Cholesterol (mg/dL) | 0.64 | 0.08 | -0.08 to 1.37 |  | -0.16 | 0.44 | -0.55 to 0.24 |  | **-0.005** | **0.95** | **-0.18 to 0.17** |  | **0.03** | **0.99** | **-26.62 to 26.67** | |
| Platelets (10^3^/µL) | 1.06 | 0.17 | -0.45 to 2.57 |  | 0.82 | 0.04 | 0.04 to 1.61 |  | **0.35** | **0.04** | **0.01 to 0.69** |  | 38.12 | 0.15 | -14.44 to 90.69 | |
| WBC (10^3^/µL) | **0.05** | **0.02** | **0.01 to 0.09** |  | 0.02 | 0.10 | -0.004 to 0.041 |  | 0.01 | 0.06 | -0.0003 to 0.019 |  | 0.90 | 0.23 | -0.59 to 2.39 | |
| Vitamin D (ng/dL) | -0.01 | 0.94 | -0.37 to 0.35 |  | -0.17 | 0.12 | -0.39 to 0.05 |  | -0.06 | 0.22 | -0.16 to 0.04 |  | -3.77 | 0.62 | -18.69 to 11.14 | |
| CRP (mg/dL) | 0.003 | 0.67 | -0.01 to 0.02 |  | 0.006 | 0.13 | -0.002 to 0.014 |  | 0.003 | 0.05 | -0.0004 to 0.007 |  | 0.49 | 0.07 | -0.03 to 1.01 | |
|  |  |  |  |  |  |  |  |  |  |  |  |  |  |  |  | |
|  |  |  |  |  |  |  |  |  |  |  |  |  |  |  |  | |
|  | **Neck (cm)** | | |  | **BMI (Kg/m^2^)** | | |  | **Waist (cm)** | | |  | **Waist to Height Ratio** | | |  |
| Parameters ^*^ | *OR* | *p-value* | *C.I. (95%)* |  | *OR* | *p-value* | *C.I. (95%)* |  | *OR* | *p-value* | *C.I. (95%)* |  | *OR* | *p-value* | *C.I. (95%)* |  |
|  |  |  |  |  |  |  |  |  |  |  |  |  |  |  |  |  |
| *Dichotomic Variables ^ψ^* |  |  |  |  |  |  |  |  |  |  |  |  |  |  |  |  |
|  |  |  |  |  |  |  |  |  |  |  |  |  |  |  |  |  |
| Snoring | **1.17** | **<0.001** | **1.11 to 1.25** |  | **1.10** | **<0.001** | **1.06 to 1.13** |  | **1.04** | **<0.001** | **1.03 to 1.05** |  | **341.58** | **<0.001** | **45.94 to 2539.82** |  |
| Smoking | 1.03 | 0.29 | 0.97 to 1.09 |  | 1.01 | 0.45 | 0.98 to 1.04 |  | 1.00 | 0.38 | 0.99 to 1.02 |  | 2.50 | 0.39 | 0.31 to 19.93 |  |
|  |  |  |  |  |  |  |  |  |  |  |  |  |  |  |  | |

^§^ Multivariate Linear Regression Model; ^ψ^ Multivariate Logistic Regression Model.

^#^ Adjusted for Age and Gender.

^*^ Abbreviations: β, coefficient; C.I., Coefficient Interval at 95%; OR, Odds Ratios; BMI, Body Mass Index; IMT, Intima-media thickness; DBP, Diastolic Blood Pressure; SBP, Systolic Blood Pressure; FBG, Fasting Blood Glucose; HDL, Hight

Density Lipoprotein; HOMA-IR, Homeostasis Model Assessment-Insulin Resistance; LDL, Low Density Lipoprotein; WBC, White Blood Cell; CRP, C-Reactive Protein.
